# Increasing Incidences and Clonal Diversity of Methicillin-Resistant *Staphylococcus aureus* in the Nordic Countries - Results From the Nordic MRSA Surveillance

**DOI:** 10.3389/fmicb.2021.668900

**Published:** 2021-04-30

**Authors:** Andreas Petersen, Kjersti W. Larssen, Frode W. Gran, Hege Enger, Sara Hæggman, Barbro Mäkitalo, Gunnsteinn Haraldsson, Laura Lindholm, Jaana Vuopio, Anna Emilie Henius, Jens Nielsen, Anders R. Larsen

**Affiliations:** ^1^Bacteria, Parasites and Fungi, Statens Serum Institut, Copenhagen, Denmark; ^2^Department of Medical Microbiology, St. Olav Hospital, Trondheim, Norway; ^3^Public Health Agency of Sweden, Solna, Sweden; ^4^Department of Clinical Microbiology, Landspitali University Hospital and University of Iceland, Reykjavik, Iceland; ^5^Finnish Institute for Health and Welfare, Helsinki, Finland; ^6^Institute of Biomedicine, University of Turku, Turku, Finland; ^7^Clinical Microbiology Laboratory, Turku University Hospital, Turku, Finland; ^8^Infectious Disease Epidemiology & Prevention, Statens Serum Institut, Copenhagen, Denmark

**Keywords:** MRSA, surveillance, *Staphylococcus aureus*, *spa*-typing, epidemiology

## Abstract

Methicillin-resistant *Staphylococcus aureus* (MRSA) is notifiable in Denmark, Finland, Iceland, Norway and Sweden. The prevalence of MRSA in this region has been low for many years, but all five countries experience increasing numbers of new cases. The aim of the study was to describe the molecular epidemiology in the Nordic countries 2009-2016. Numbers of new cases of MRSA from 1997 to 2016 were compared, and a database containing information on *spa*-type and place of residence or acquisition, for all new MRSA isolates from 2009 to 2016 was established. A website was developed to visualize the geographic distribution of the *spa*-types. The incidence of new MRSA cases increased in all Nordic countries with Denmark having 61.8 new cases per 100,000 inhabitants in 2016 as the highest. The number of new cases 2009 to 2016 was 60,984. *spa*-typing revealed a high genetic diversity, with a total of 2,344 different *spa*-types identified. The majority of these *spa*-types (N = 2,017) were found in 1-10 cases. The most common *spa*-types t127/CC1, t223/CC22, and t304/CC6:8 increased significantly in all Nordic countries during the study period, except for Iceland, while *spa*-type t002/CC5 decreased in the same four countries. The trends of other common *spa*-types were different in each of the Nordic countries. The Nordic countries were shown to share similar trends but also to have country-specific characteristics in their MRSA populations. A continued increasing numbers of MRSA will challenge the surveillance economically. A more selected molecular surveillance will probably have to be employed in the future.

## Introduction

The Nordic countries (Denmark, Finland, Iceland, Norway and Sweden) have had a very low prevalence of methicillin resistant *Staphylococcus aureus* (MRSA) for many years, with <2.5% of *S. aureus* bacteraemia isolates being MRSA (Surveillance Atlas of Infectious Diseases)^1^. This is probably due to several factors such as a prudent use of antibiotics and a longstanding extensive MRSA screening, surveillance and infection control programs, outlined in regional and national guidelines with the main points summarized in Table 1. Public funding of costs related to screening, follow up and treatment and/or decolonization may also be important factors in keeping MRSA transmission rates at a low level.

**TABLE 1:** Health care systems and MRSA policy in the Nordic countries.

**Country**	**Sweden**	**Denmark**	**Finland**	**Norway**	**Iceland**
**Health care systems**					
Assigned family doctor	Yes	Yes	No	Yes	Yes
Government financed care	Free/substantially sponsored	Free	Substantially sponsored	Free/substantially sponsored^§^	Free/substantially sponsored^§^
**MRSA policy**					
Infection notified	Since 2000	Since 2006	Since 1995	Since 1995	Since 2008
Carriage notified	Since 2000	Since 2006	Since 1995	Since 2005	Since 2008
MRSA screening	At hospital admission or according to risk factors*	At hospital admission or before work according to risk factors*	At hospital admission or according to risk factors*	At hospital admission or before work according to risk factors*	At hospital admission or according to risk factors*
Contact tracing in HCI	Unexpected finding in patient	Unexpected finding in patient	Unexpected finding in patient	Unexpected finding in patient or HCW	Unexpected finding in patient
Contact tracing in PC	Household members or in relation to outbreaks	Household members or in relation to outbreaks	Depending on risk factors^#^	Depending on risk factors^#^ or in relation to outbreaks	Depending on risk factors^#^ or in relation to outbreaks
Isolation in hospitals	Single room if available	Contact isolation in single room	Contact precautions	Contact isolation in single room	Contact isolation in single room
Isolation in NHs	Standard and contact precautions (patient depending)	Single room with barrier precautions	Contact or standard precaution (unit and patient depending)	Contact or standard precaution (unit and patient depending)	Single room No isolation
Work restrictions	Depending on risk factors^&^	H work allowed if decolonized Relocation considered	H work allowed if decolonized and no skin problems Relocation considered if skin lesions/problems	Clinical work in HCI not allowed	Clinical work in HCI not allowed
Decolonization therapy for HCWs	Selected cases	Should be offered	Should be offered	Should be offered	Always
Decolonization therapy for others	Selected cases	Offered to carriers and households, except if working with live pigs	Offered to patients, in outbreak situations and all pre- surgery	Offered to patients. Recommendation depends on risk factors	Most patients. Recommendation depends on risk factors
Treatment free of charge?	Yes	Infections are subsidized. Carriage is free in 4/5 regions	Infections and carriage are subsidized. HCW paid by employer (hospital)	Infections and carriage	Infections and carriage
Follow up of HCWs	Until 3 negative samples in a one-year period.	At day 1, 7, 14, 21 + 6 months.	After decolonization: three controls and if hospitalized at admission	At 1,2 and 3 weeks + 3,6 and 12 months	At 1, 2 and 3 weeks + 3, 6 and 12 months
Follow up of other carriers	Until 3 negative samples in a one-year period	Hs: At 1, 2 and 3 weeks + 6 months. PC: At 1 + 6 months	At readmission. After decolonization: three controls	At 1,2 and 3 weeks + 3,6 and 12 months	Depending on risk factors

*Abbreviations: HCI = Health Care Institution, H = Hospital, NH = Nursing home, PC = Primary care; HCW = Health care worker, S = Sweden, D = Denmark, F = Finland, N = Norway, I = Iceland.*

*^§^ Free for children and inpatients.*

^#^ Family and work related risk for transmission is evaluated.

*Common Nordic risk factors: Close contact with known MRSA carrier (household), Hospital admission outside Nordic countries, or Work in HCI, refugee camp or orphanage outside Nordic countries the past 6-12 months.

* Country unique risk factors; D: Work with live pigs (including household members). F and N: Patient transfer from an MRSA epidemic ward or endemic long-term unit/hospital. I: Having recurrent skin infections. Being an asylum seeker or a refugee. D and I: Previous MRSA positive (6 months). N: Previous MRSA positive and not three consecutive negative MRSA samples.

*^&^ Risk factors for MRSA transmission. Negative cultures may be required in high risk wards.*

MRSA is listed as a notifiable finding in the Nordic countries, and the countries have well established national surveillance systems for MRSA. The reference laboratories contribute with genotyping of the isolates. Since 2006-2009, the surveillance in the Nordic countries has included *spa*-typing, detection of Panton-Valentine leukocidin (PVL) genes as well as registration of clinical and/or demographic data. The Nordic Staphylococci Reference Laboratories (NSRL) share information on the MRSA surveillance data and methodology and quality control programs, with an overall aim to provide the best laboratory methods in place for the surveillance to support the control of MRSA especially in hospitals, and keeping the prevalence of invasive MRSA low (Skov and SSAC MRSA Working Party, 2005; Skov et al., 2008).

An increase in the numbers of new MRSA cases and a shift toward more cases of community acquired MRSA have been observed in the Nordic countries (Stenhem et al., 2006; Larsen et al., 2009, Holzknecht et al., 2010; Elstrom et al., 2012; Larsson et al., 2014; Swedres-Svarm, 2016; Junnila et al., 2020). This challenges the infection control practices and actions to prevent further transmission, and although the majority of cases are asymptomatic carriers, found through screening and contact tracing, or uncomplicated skin or soft tissue infections, an increase in MRSA blood stream infections or other severe infections may be expected in the years to come (Datta and Huang, 2008).

The aim of this study was to compare the emergence and dissemination of MRSA clones in the Nordic countries. This was done by assembling *spa*-typing results and associated demographic data in one database. Data are visualized by the use of a web based Geographic Information System (GIS-mapping) tool. The tool was developed to ensure a freely accessible and searchable database, which can easily visualize differences and trends in emerging MRSA clones within or between countries. The increasing dataset provides insight into clones of particular interest that may help guide national surveillance systems and policy makers in directing measures to control further spread of MRSA.

## Materials and Methods

The number of MRSA cases reported to the NSRLs between 1997 and 2016 were compared with population data to obtain MRSA incidence in the respective countries. Demographic data were retrieved from the following sources: Statistics Denmark^2^, Statistics Finland^3^, Statistics Iceland^4^, Statistics Norway^5^, and Statistics Sweden^6^.

Since 2009, the Nordic countries use the same typing methods for national surveillance of MRSA. Relevant genetic information such as *spa*-type, clonal complex (CC), presence of PVL-gene, and date (of MRSA sample taken or received) were uploaded to a central Nordic database and linked to geolocation representing where and when the isolate was found.

Frequency of the most common *spa*-types and PVL-positive isolates seen in relation to the total number of isolates was calculated for each year. Negative binomial regression analysis was used to analyze for trends for *spa*-types and prevalence of PVL-positive MRSA for each country. Trends were described by incidence rate ratios (IRR).

Simpson’s index of diversity of *spa*-types was calculated for all isolates and for each country (Hunter and Gaston, 1988).

A website was developed to illustrate the geographical distribution of the MRSA isolates^7^. Nordicmrsa.org provides a graphical user interface utilizing Leaflet (Cheng et al., 2019) to create a dynamic map that takes latitude and longitude to display the geolocation of selected *spa*-types or CC with customized markers on the map. The app has been implemented in R using the *shiny* package (Chang et al., 2020; R Core Team, 2020).

### Ethical Considerations

This publication only made use of bacterial isolates and typing results submitted to the reference laboratories as part of national MRSA surveillance programs. Cases were anonymized by each NSRL. No patient information was used or shared between the research group members. Geolocation are shown on an aggregated level in order to ensure anonymity represented by either hospital district-level (Finland and Iceland), county-level (Sweden), hospital or city hall, depending on whether the isolates were found within a hospital or in the community (Denmark) or at a municipality-level (Norway).

## Results

**FIGURE 1:**
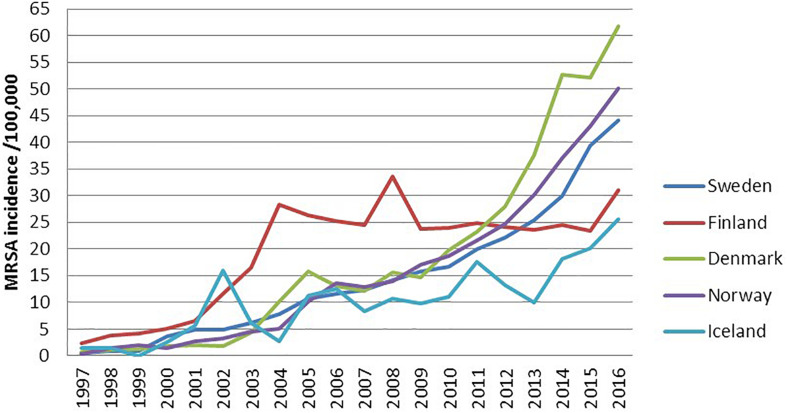
Incidence of notified MRSA cases in the Nordic countries, 1997–2016.

Before year 2000, the Nordic countries had very low numbers of MRSA cases, which increased steadily throughout the study period. From 2000 to 2016 the annual incidence rates of MRSA increased 6-35 fold, except in Finland where it remained relatively stable after 2004, between 23.7 and 33.5 cases per 100.000 inhabitants (Figure 1). Denmark experienced a particular high incidence increase from 2012, reaching the highest incidence of 61.8 in 2016. This was largely due to the epidemic of livestock associated (LA-) MRSA CC398 comprising 35% of all new Danish MRSA cases in 2016 (Figure 1). LA-MRSA CC398 mostly consists of *spa*-types t034 and t011. LA-MRSA CC398 were only encountered in a limited number of cases in the other Nordic countries during the study period (*N* = 2, 97, 81 and 113 for Iceland, Sweden, Finland and Norway, respectively). When excluding LA-MRSA CC398, the increase in Denmark was similar to the ones in Sweden and Norway, reaching incidences of 40.1, 44.0, and 50.1 cases per 100,000 inhabitants, respectively.

The population in the Nordic countries ranged from 332,526 in Iceland to 9,851,017 in Sweden in 2016. All Nordic countries experienced a population increase of 3.0-8.6% between 2009 and 2016. A total of 60,984 new MRSA cases were reported in the Nordic countries in 2009-16. The total numbers for each country were: 20,683; 16,234; 11,320; 12,339 and 408 for Sweden, Denmark, Finland, Norway and Iceland, respectively.

*spa*-typing revealed a very high genetic diversity, with a total of 2,344 different types identified. Of these 2,017 were observed in ≤10 cases across the period, hereafter referred to as sporadic clones. A total of 646 (1.1%) isolates were non-typeable with *spa*-typing. The diversity increased from 485 different *spa*- types in 2009 to 910 in 2016 (excluding CC398 isolates from Denmark). Sweden and Norway had the highest diversity, but a pattern with a high number of *spa*-types identified only in one year, and a low number of *spa*-types identified in all years, was observed in all Nordic countries (Table 2). Of the many different *spa*-types found in each of the countries, between 54-65% were found in only one of the years in the study period (Table 2). In contrast, only 2-6% of the *spa*-types in each country were found throughout the period. In Norway, Denmark, Finland and Sweden between 37-46% of all *spa*-types were only found in the respective country (Table 2). For Iceland 13% of the *spa*-types were not found in any of the other Nordic countries.

**TABLE 2:** Diversity of *spa-*types among MRSA in the Nordic countries 2009-2016.

**Country**	**Denmark**	**Finland**	**Iceland**	**Norway**	**Sweden**	**All**
No of *spa*- types	890	709	91	904	1266	2344
No of *spa*- types occurring only in one year	522 (59%)	409 (58%)	59 (65%)	499 (55%)	689 (54%)	1248 (53%)
No of *spa-* types occurring in all years	42 (5%)	35 (5%)	2 (2%)	44 (5%)	70 (6%)	125 (5%)
No of *spa*-types unique for the country	332 (37%)	286 (40%)	13 (14%)	321 (36%)	593 (47%)	NA
Simpson’s Diversity Index	0.95	0.93	0.94	0.97	0.97	0.97

*NA: Not applicable*

**FIGURE 2:**
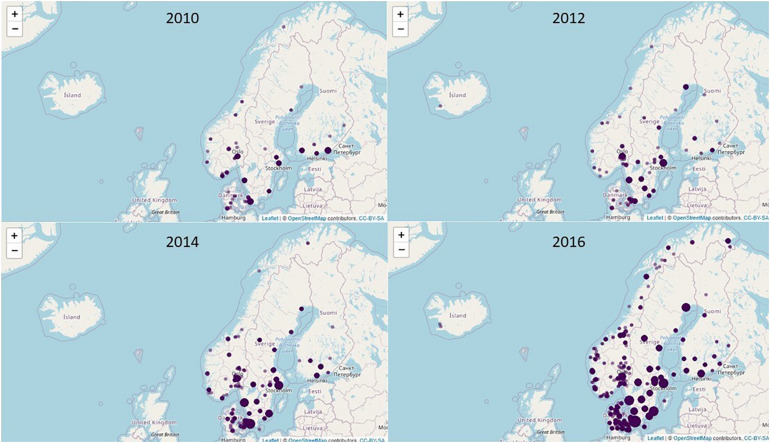
Geographic distribution of MRSA *spa*-type t223 in the Nordic countries in the years.2010, 2012, 2014, and 2016.

Table 3 shows trends during the study period for the ten most prevalent *spa*-types in the Nordic countries. The few number of MRSA isolates in Iceland prevented any statistical analysis of trends. For the remaining countries, similarities and dissimilarities were seen. The proportion of *spa*-types t127/CC1, t223/CC22 and t304/CC6:8 increased significantly, with an incidence rate ratio (IRR) between 1.10 – 1.19 for t127, 1.17 – 1.21 for t223, and 1.25 – 1.51 for t304. In contrast, *spa*-type t002 decreased in prevalence in the four Nordic countries. For other *spa*-types the trends were different in each of the four Nordic countries; as illustrated by t044/CC80 which decreased in prevalence in Denmark and Norway, was stable in Sweden, and increased in Finland (Table 3). Figure 2 shows the geographic distribution of *spa*-type t223/CC22 in the Nordic countries in the years 2010, 2012, 2014, and 2016, respectively. From 149 cases in 2010, the numbers increased and was 1,024 in 2016.

**TABLE 3:** Numbers and trends of the ten most prevalent *spa* types among MRSA cases in the Nordic countries in 2009–2016. Total denotes total number of MRSA per year and trend of total numbers.

**Year**										
**Country/spa type/Kreiswirth**	**2009**	**2010**	**2011**	**2012**	**2013**	**2014**	**2015**	**2016**	**Incidence Rate Ratio (IRR) (95% confidence interval)**	**P**
**Sweden**										
t223/CC22/TJEJCMOMOKR	46	54	76	106	153	232	332	501	1.21 (1.18 – 1.25)	0.000
t008/CC8/YHGFMBQBLO	151	149	168	133	187	187	185	173	0.88 (0.85 – 0.91)	0.000
t002/CC5/TJMBMDMGMK	101	101	169	176	178	173	206	203	0.94 (0.89 – 0.98)	0.008
t044/CC80/UJGBBPB	105	98	92	81	124	179	271	225	0.99 (0.94 – 1.05)	0.797
t304/CC6:8/YC2FMBQBLO	11	20	22	46	64	120	234	511	1.49 (1.40 – 1.59)	0.000
t127/CC1/UJFKBPE	44	51	70	68	121	128	233	249	1.11 (1.07 – 1.15)	0.000
t019/CC30/XKAKAOMQ	59	65	131	107	93	116	90	100	0.89 (0.82 – 0.97)	0.007
t690/CC88/UGFMEEBBBPB	10	21	46	38	50	72	110	94	1.12 (1.03 – 1.21)	0.005
t437/CC59/ZDMDMOB	52	51	49	56	50	69	50	48	0.85 (0.82 – 0.89)	0.000
t386/CC1/UJE	4	9	6	16	32	51	95	107	1.36 (1.26 – 1.48)	0.000

Total	**1478**	**1580**	**1881**	**2096**	**2451**	**2919**	**3880**	**4398**	**1.18**	**0.000**

**Denmark**										
t034/CC398/XKAOAOBQO	27	93	130	185	527	972	(237)^a^	(118)^a^	1.51^b^ (1.40 – 1.63)	0.000
t002/CC5/TJMBMDMGMK	88	110	119	141	161	175	172	188	0.89 (0.87 – 0.92)	0.000
t008/CC8/YHGFMBQBLO	90	92	122	130	113	114	122	106	0.83 (0.79 – 0.86)	0.000
t019/CC30/XKAKAOMQ	43	73	97	97	92	67	89	96	0.86 (0.80 – 0.92)	0.000
t127/CC1/UJFKBPE	13	33	46	43	87	130	148	135	1.10 (1.03 – 1.17)	0.003
t304/CC6:8/YC2FMBQBLO	1	5	37	74	75	76	111	224	1.36 (1.09 – 1.71)	0.008
t223/CC22/TJEJCMOMOKR	16	17	29	37	47	95	87	212	1.18 (1.10 – 1.27)	0.000
t044/CC80/UJGBBPB	61	36	58	45	47	37	48	84	0.84 (0.77 – 0.91)	0.000
t024/CC8/YGFMBQBLO	66	80	68	43	42	44	26	15	0.67 (0.64 – 0.71)	0.000
t032/CC22/TJJEJNF2MNF2MOMOKR	24	39	33	88	49	68	41	26	0.83 (0.72 – 0.95)	0.008

Total	**814**	**1094**	**1291**	**1556**	**2091**	**2872**	**2967**	**3549**	**1.27**	**0.000**

**Finland**										
t067/CC5/TJMBMDMGM	348	370	382	209	217	141	75	144	0.80 (0.75 – 0.85)	0.000
t172/CC59/ZDMA3KB	211	189	257	228	233	259	214	188	0.98 (0.93 – 1.03)	0.37
t008/CC8/YHGFMBQBLO	100	120	127	165	143	150	114	219	1.04 (1.00 – 1.09)	0.04
t002/CC5/TJMBMDMGMK	63	55	62	58	39	44	49	60	0.95 (0.91 – 0.99)	0.012
t032/CC22/TJJEJNF2MNF2MOMOKR	74	42	39	44	52	43	22	18	0.85 (0.78 – 0.93)	0.000
t044/CC80/UJGBBPB	17	17	14	32	46	38	47	64	1.19 (1.11 – 1.28)	0.000
t127/CC1/UJFKBPE	18	13	24	19	36	26	50	64	1.19 (1.12 – 1.27)	0.000
t223/CC22/TJEJCMOMOKR	10	37	18	15	21	26	43	77	1.17 (1.04 – 1.30)	0.008
t304/CC6:8/YC2FMBQBLO	4	4	17	14	19	11	52	123	1.51 (1.32 – 1.73)	0.000
t020/CC22/TJNF2MNF2MOMOKR	33	29	16	22	24	41	26	32	1.00 (0.92 – 1.08)	0.945

Total	**1331**	**1328**	**1442**	**1355**	**1330**	**1389**	**1348**	**1797**	**1.03**	**0.032**

**Norway**										
t002/CC5/TJMBMDMGMK	92	100	125	135	154	177	236	221	0.97 (0.95 – 1.00)	0.021
t019/CC30/XKAKAOMQ	59	82	135	124	157	151	150	100	0.92 (0.85 – 1.00)	0.058
t008/CC8/YHGFMBQBLO	91	84	100	79	98	121	139	112	0.90 (0.87 – 0.92)	0.000
t223/CC22/TJEJCMOMOKR	20	41	44	49	78	90	176	232	1.18 (1.13 – 1.24)	0.000
t127/CC1/UJFKBPE	15	37	25	52	63	112	114	168	1.16 (1.09 – 1.22)	0.000
t044/CC80/UJGBBPB	47	53	69	43	26	77	80	85	0.91 (0.84 – 0.99)	0.033
t304/CC6:8/YC2FMBQBLO	11	17	31	20	33	51	98	202	1.25 (1.14 – 1.37)	0.000
t437/CC59/ZDMDMOB	25	21	35	40	44	60	48	80	1.00 (0.95 – 1.05)	0.980
t657/CC97/TJEFMBPB	5	11	12	9	25	48	35	44	1.14 (1.03 – 1.27)	0.012
t688/CC5/TJMBMK	21		28	16	13	31	39	34	0.98 (0.80 – 1.20)	0.852

Total	**817**	**958**	**1141**	**1219**	**1546**	**1853**	**2271**	**2534**	**1.18**	**0.000**

**Iceland**										
t019/CC30/XKAKAOMQ	1	8	14	8	8	3	11	19	1.04 (0.89 – 1.20)	0.628
t008/CC8/YHGFMBQBLO	5	6	6	4	11	8	8	4	0.93 (0.80 – 1.07)	0.296
t002/CC5/TJMBMDMGMK	3	1	1	3	2	5		5		
t186/CC88/UGFMEEBBPB		3	1	3		3	5	2		
t253/CC30/WGKAKAOMQQQQ							15			
t437/CC59/ZDMDMOB	1	2	10			1		1		
t012/CC30/WGKAKAOMQQ	3	1	4	1			4			
t127/CC1/UJFKBPE		1		1	3	1	1	5		
t1228/NR/TJMGMDMGMK						12				
t3364/CC8/YHGFMFBQBLO		2						6		

Total	**33**	**36**	**58**	**42**	**35**	**58**	**66**	**80**	**1.12**	**0.000**

*a) Only a minor fraction of CC398 were spa typed after 2014 so the numbers are not representative b) IRR calculated for years 2009-2014. NR:not resolved*

Country-specific clones included the livestock-associated t034/CC398, t011/CC398 and the *mecC* -MRSA t843/CC130 in Denmark, and t067/CC5 and t172/CC59 in Finland. Seventy percent of the *spa*-types t790/CC22 and t991/CC913 were found in Sweden.

Information on PVL was available for 50,313 isolates (83%). The information was almost complete for Denmark, Iceland, Norway and Sweden (96-100%) while Finland only had information for 1,489 isolates (13%). A total of 16,175 isolates were PVL-positive (32%) with a decreasing trend during the study period (36% in 2009, 28% in 2016; IRR = 0.95 (0.94-0.97)). Table 4 shows the ten most prevalent PVL-positive *spa*-types. The three most prevalent were t008/CC8, which corresponds to the USA 300 clone, t019/CC30, (South-West Pacific clone) and t044/CC80, the European community-acquired (CA)-MRSA clone.

**TABLE 4:** The ten most prevalent PVL positive MRSA *spa* types in the Nordic countries 2009-2016 in total and by year.

***spa* type**	**Number of PVL positive/% of total tested**	**2009**	**2010**	**2011**	**2012**	**2013**	**2014**	**2015**	**2016**
t008/CC8/YHGFMBQBLO	2696/82	289	296	334	299	381	392	380	325
t019/CC30/XKAKAOMQ	2408/96	164	227	365	341	361	341	319	290
t044/CC80/UJGBBPB	2052/96	223	190	216	177	200	295	385	366
t002/CC5/TJMBMDMGMK	1149/31	65	86	121	181	163	157	182	194
t437/CC59/ZDMDMOB	794/74	63	73	92	84	104	130	109	139
t657/CC97/TJEFMBPB	520/97	27	45	62	56	59	84	87	100
t690/CC88/UGFMEEBBBPB	451/63	4	27	48	35	40	74	120	103
t127/CC1/UJFKBPE	399/18	6	9	12	9	59	74	127	103
t852/CC22/UJEJNCMOMOKR	273/95	24	19	18	36	42	34	47	53
t021/CC30/WGKAKAOMQ	272/68	6	19	15	19	28	27	63	95

## Discussion

In our comparison of MRSA surveillance results across the Nordic Countries, we have observed several uniform patterns, including an overall low prevalence of MRSA, but also a rapidly increasing incidence in the last decade.

The low prevalence of MRSA in the Nordic countries likely results from the use of preventive guidelines established more than a decade ago with the main aim of protecting in-patients. A European comparison of guidelines for MRSA prevention and control pointed out that having longstanding MRSA prevention guidelines that also included nursing homes, general practice and home care, as well as notification of MRSA and isolation of MRSA cases in single rooms, were among the features that separated low prevalent MRSA countries from countries with higher MRSA prevalence (Kalenic et al., 2010).

The increase of MRSA in the Nordic countries was driven by increases outside hospital settings by both LA- and CA-MRSA (Di Ruscio et al., 2018; Junnila et al., 2020). The typical PVL-positive CA-MRSA clones (e.g., CC8, CC30, and CC80) are established in the community and are most likely supplemented with new imported cases due to travel and immigration. Several reports have highlighted increasing travel to and migration from high- prevalence countries and subsequent spread in the communities as a major route for the increasing number of MRSA in the Nordic countries. This assumption has been supported by results obtained by routine typing, and detection of the PVL genes of all MRSA (Fossum and Bukholm, 2006; Stenhem et al., 2006; Böcher et al., 2008; Kanerva et al., 2009; Larsen et al., 2009; Holzknecht et al., 2010; Vainio et al., 2011; Larsson et al., 2014; Nurjadi et al., 2015). However, in contrast to this assumption the overall prevalence of PVL-positive MRSA was decreasing in the Nordic countries during the study period.

The total number of MRSA reported here comprises both infections and screening samples. Screening practices have varied through the study period and, to some extent, between the Nordic countries. However, when looking only at the blood stream infections, an increase has also been observed (see Text Footnote 1), suggesting that an increased burden of MRSA may lead to more serious infections.

The genetic diversity of MRSA in the Nordic countries was very high with the vast majority of *spa*-types being just sporadic, causing very limited spread. Only a few MRSA clones seem to prevail, and these are in general international successful MRSA clones.

We observed a recent increase in the number of t223/CC22 and t304/CC6:8 isolates, which are prevalent in the Middle East countries, indicating that this may be due to immigration related to the civil war in Syria (Aro and Kantele, 2018). *spa*-type t223/CC22 (mainly PVL negative) has been observed in both community and health care settings in Norway, primarily as colonization, and largely associated with immigration from Syria, Russia and Afghanistan in recent years (Di Ruscio et al., 2018). In Sweden, this *spa*-type emerged rapidly around 2010, and was associated with young age and increased immigration from the Middle East (Larsson et al., 2014).

Norway experienced several hospital acquired MRSA (HA-MRSA) outbreaks of PVL negative t304/CC8 in South Western Norway between 2004 and 2011. Since then this specific clone has become extinct, and replaced by other t304 clones more associated with the community setting (Blomfeldt et al., 2016; Di Ruscio et al., 2018). In Denmark, t304/CC6 has been associated with hospital outbreaks in recent years (Bartels et al., 2015).

In addition to clones demonstrated among several of the Nordic countries, some country-specific differences were noted. Since 2009, dissemination of LA-MRSA CC398 in Danish pigs has been a main driver of the increase in new MRSA cases. Denmark differs from the other Nordic countries by having intensive pig farming, with more than 30 million pigs produced annually and approximately 10,000 people working in the farms. An investigation in 2016 found 88% of the pig farms to be MRSA positive (DANMAP, 2016). The annual number of new human LA-MRSA CC398 cases in 2014-2016 was between 1,173-1,277 with 85% having direct contact to pigs (DANMAP, 2016). In contrast, Norway has a less intensive pig production with approximately 1.6 million pigs slaughtered annually. After several separate introductions of LA-MRSA in 2013-2014, a strict policy including a yearly surveillance program, eradication of MRSA in stables by slaughter of pigs and decontamination of personnel has been applied (Grøntvedt et al., 2016). This strategy has so far proven successful, with no cases identified in 872 screened pig herds in Norway in 2016. In the other Nordic countries, spread in pigs and humans has so far been sporadic.

*spa*-type t067/ST125/CC5/SCC*mec* IA, PVL negative was involved in a large HA-associated outbreak mainly in one of the hospital districts from Finland. It affected both long-term care facilities and acute hospitals during 2001-2011 (Kanerva et al., 2009; Laine et al., 2013; Jokinen et al., 2015), but decreased from 350-400 cases in the beginning to 75 cases in 2015. An increase was again recorded in 2016 (N = 144). *spa*-type t172/ST375, CC59, SCC*mec* IV, PVL negative was almost exclusively found in Finland (1,779/1,882, 94%). It is the most common CA-MRSA in Finland, but it is not known why it is almost absent in the other Nordic countries. *spa*-type t991/CC913 was primarily found in Sweden (236/337, 70%). It was involved in outbreaks in hospital settings involving 25 cases, but no major explanation for the country-specificity of this clone in Sweden could be established. One third of the patients originated from the Middle East.

In Norway, the PVL-positive, multi-resistant Bengal Bay clone t657/ST772/CC97/SCC*mec* V, PVL positive has increased rapidly and caused several small-scale outbreaks in South Eastern Norway, particularly since 2013-2014. Cases have been linked mainly to the Indian subcontinent, through family history, home country visit, tourism and immigration (Blomfeldt et al., 2017).

*spa* typing is the typing method of choice in all Nordic countries. It is rapid, unambiguous and the *spa* types are easily communicated. It can be used in routine infection control in hospitals as well as describing national and international trends. s*pa* types of MRSA isolated from individual patients are also surprisingly stable over time, given that single mutations or genetic alterations change the *spa* type. For the most prevalent types, additional typing such as MLST and SCC*mec* may be useful to characterize the isolates in more depth. Whole genome sequencing has been implemented in most of the NSRLs to be used for expanded typing and characterization of strains of interest, either for outbreak investigations, epidemiological studies and/or primary research.

The MRSA policies in the Nordic countries seems rather effective in preventing MRSA in hospitals, but have only limited effect on CA-MRSA and import of MRSA. MRSA imported by travel and migration spread in the community and can explain part of the increase observed both in total numbers of registered cases, and in the increasing diversity of clones. In line with this, a recent study reported import of travel-associated CA-MRSA to Europe from intercontinental travelers with skin and soft-tissue infections and found that the condition was transmissible (Nurjadi et al., 2019).

The increasing numbers of MRSA and other antimicrobial resistant microorganisms will continue to challenge the Nordic health care systems and call for revisions of the established policies. The NSRLs play an important role in providing high quality surveillance data for infection control and decision making, in a timely and easy-to-reach manner. In this study we have developed and combined our data in a publicly available database and used it to describe common traits and new emerging threats in the Nordic countries. This has only been possible due to the political and methodological similarities in our countries and can therefore not easily encompass other countries. The NSRLs are now challenged economically by the increase in number of MRSA cases. In the future, a more selective genotyping based on risk factors and/or selected epidemiological settings, such as association to outbreaks, livestock, invasive infections or health-care institutions will probably have to be employed. *spa*-typing is, due to the large genetic diversity still an inexpensive and effective screening method for epidemiological surveillance but whole genome sequencing is needed to investigate outbreaks and new transmission patterns. Studies like the current will therefore be difficult to repeat in the future, as the NRLs in the Nordic countries will have different priorities.

## Data Availability Statement

The raw data supporting the conclusions of this article will be made available by the authors, without undue reservation.

## Author Contributions

AP conceived the study, collected and analyzed the data, and wrote and revised the manuscript. KL conceived the study, collected the data, and wrote the manuscript. FG collected the data, set up the home page, and revised the manuscript. HE, BM, and JV collected the data and revised the manuscript. SH, LL, and GH conceived the study, collected the data, and revised the manuscript. AH wrote the app for displaying MRSA cases and revised the manuscript. JN performed the statistical analysis and revised the manuscript. AL conceived the study and wrote and revised the manuscript. All authors contributed to the article and approved the submitted version.

## Conflict of Interest

The authors declare that the research was conducted in the absence of any commercial or financial relationships that could be construed as a potential conflict of interest.

## Footnotes

1 https://atlas.ecdc.europa.eu/public/index.aspx

2 www.dst.dk

3 www.stat.fi

4 www.statice.is

5 www.ssb.no

6 www.scb.se

7 www.nordicmrsa.org
